# Symbiopersonal intelligence towards symbiotic and personalized digital medicine

**DOI:** 10.1016/j.fmre.2025.01.009

**Published:** 2025-01-23

**Authors:** Alfred Mensah, Qiwen Bao, Zhaonan Zhang, Ya Chen, Qing Jiang, Pingqiang Cai

**Affiliations:** a Translational Mechanomedicine Lab, Medical School, Nanjing University, Nanjing 210093, China; b Division of Sports Medicine and Adult Reconstructive Surgery, Department of Orthopedic Surgery, Nanjing Drum Tower Hospital, Nanjing 210008, China; c Nanjing Engineering Research Center for Proactive Health and Prognostic Wearables, Nanjing 210024, China

**Keywords:** Artificial intelligence, Healthcare, Wearable devices, Implantable devices, Flexible electronics, Advanced materials

## Abstract

In this perspective, we introduce the concept of Symbiopersonal Intelligence (SymAI)—a specialized form of artificial intelligence designed to facilitate and optimize symbiotic interactions between individuals and intelligent devices in digital medicine. SymAI represents a new frontier in personalized intelligent systems, adaptively learning from and catering to individual needs and behaviors. We explore its emergence and potential implementation in both personal and public healthcare, encompassing telemedicine, precision medicine, surgical assistance, chronic disease management, and policy optimization. Key technological frameworks and hardware enablers are outlined, with a particular emphasis on multimodal data retrieval, transmission, and processing, as well as personalized interventions delivered via wearable and implantable devices. By integrating artificial intelligence into sensor technologies and addressing barriers in flexible electronics, SymAI holds the potential to revolutionize digital health, offering more responsive, tailored care and improved health outcomes.

## 1 *Symbiopersonal intelligence*: redefining human-machine interactions in digital medicine

Licklider’s seminal 1960 report, “Man-Computer Symbiosis” anticipated that humans and computers would establish a symbiotic relationship through cooperative interaction [1]. Today, this vision has become a reality, evidenced by notable achievements across various sectors, including finance, e-commerce, manufacturing, education, transportation, agriculture, and healthcare—a key industry for enhancing quality of life. AI-driven personalized intelligent systems provide solutions in healthcare, education, labor-intensive tasks, and complex technologies to augment human life. These systems understand their users’ characteristics, expectations, and motivations through mutual interactions, and thus provide beneficial customized solutions. In essence, human-computer interaction involves humans interacting with machines to perform tasks and receive personalized solutions and feedback. This type of cooperation exemplifies a variant of symbiotic coexistence—specifically, the commensalistic type.

The evolution of symbiotic interactions has given rise to the exploration of artificial embodied agents with well-adapted morphologies that can learn control tasks in diverse, complex environments. Embodied intelligence is an approach to understanding and engineering intelligent behavior in embodied and situated agents by considering the strict coupling between the agent and its environment, mediated by the constraints of the agent’s own body, including perceptual, motor and computing systems. The emergence of embodied intelligence is closely linked to parallel developments in computational intelligence and robotics, focusing on morphological computation and sensorimotor coordination in evolutionary robotics models. In neuroscience and cognitive sciences, the focus is on embodied cognition and developmental robotics models of embodied symbol learning [2]. By contrast, this perspective aims to define and categorize a rapidly evolving intelligent system that features the symbiotic interaction between humans and machines within the realm of digital medicine. We endeavor to unveil a new frontier of next-generation personalized intelligent healthcare systems that we posit will facilitate intermediation between humans for mutualistic symbiotic interdependence. To gain a substantive understanding, we have coined a new and appropriate term: “symbiopersonal intelligence”.

**Fig. 1:**
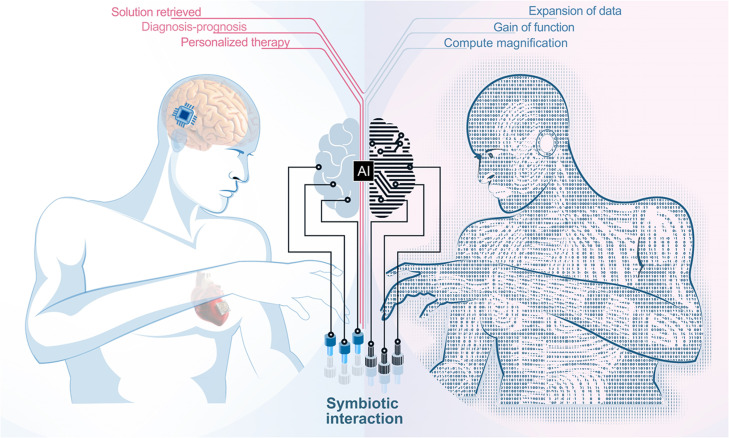
**Overview of symbiopersonal intelligence (SymAI)**.

*Symbiopersonal Intelligence* is a term that defines specialized forms of intelligence that facilitate and optimize symbiotic interactions between an individual and an attendant machine. These systems continuously learn from and adapt to personal needs and behaviors to provide adaptive healthcare solutions. In *Symbiopersonal Intelligence* (SymAI), a machine or device mediates and facilitates solution-oriented interactions between humans and machines or between humans, to mitigate defects or gaps in the interaction process (Fig. 1). The foundational principle is as follows: Symbiopersonal systems primarily utilize intelligence to enhance symbiotic interactions between individuals and systems, subsequently expanding to reveal a novel frontier, namely human-human machine-facilitated interaction. In effect, the human gleans the benefits of solution retrieval, gets diagnosis or prognosis, and receives personalized therapy from the machine, whereas the machine in turn benefits from expansion of input data, gain of function, and magnification of compute power. AI systems under this extensive canopy have the capability to create personalized, adaptive, and significantly beneficial interactions with and between humans and machine entities in real time.

The differentiating feature of SymAI that marks its unique distinction in relation to existing (general) artificial intelligence is this: existing artificial intelligence systems are unilaterally implemented to provide solutions to human needs by leveraging their pre-programmed unique strengths and capabilities, whereas SymAI systems are ‘exclusively personal’ intelligent agents that abide on, with, or in their human counterpart engaged in a bidirectional interactive exchange of (adaptive) data or solutions on both ends. This is furthermore different from intelligent medicine or artificial intelligence in medicine. Artificial intelligence in medicine is the use of machine learning models to help process medical data, give medical professionals important insights, and improve health outcomes and patient experiences.

## 2 Implementations of SymAI in personal healthcare and public health

**Fig. 2:**
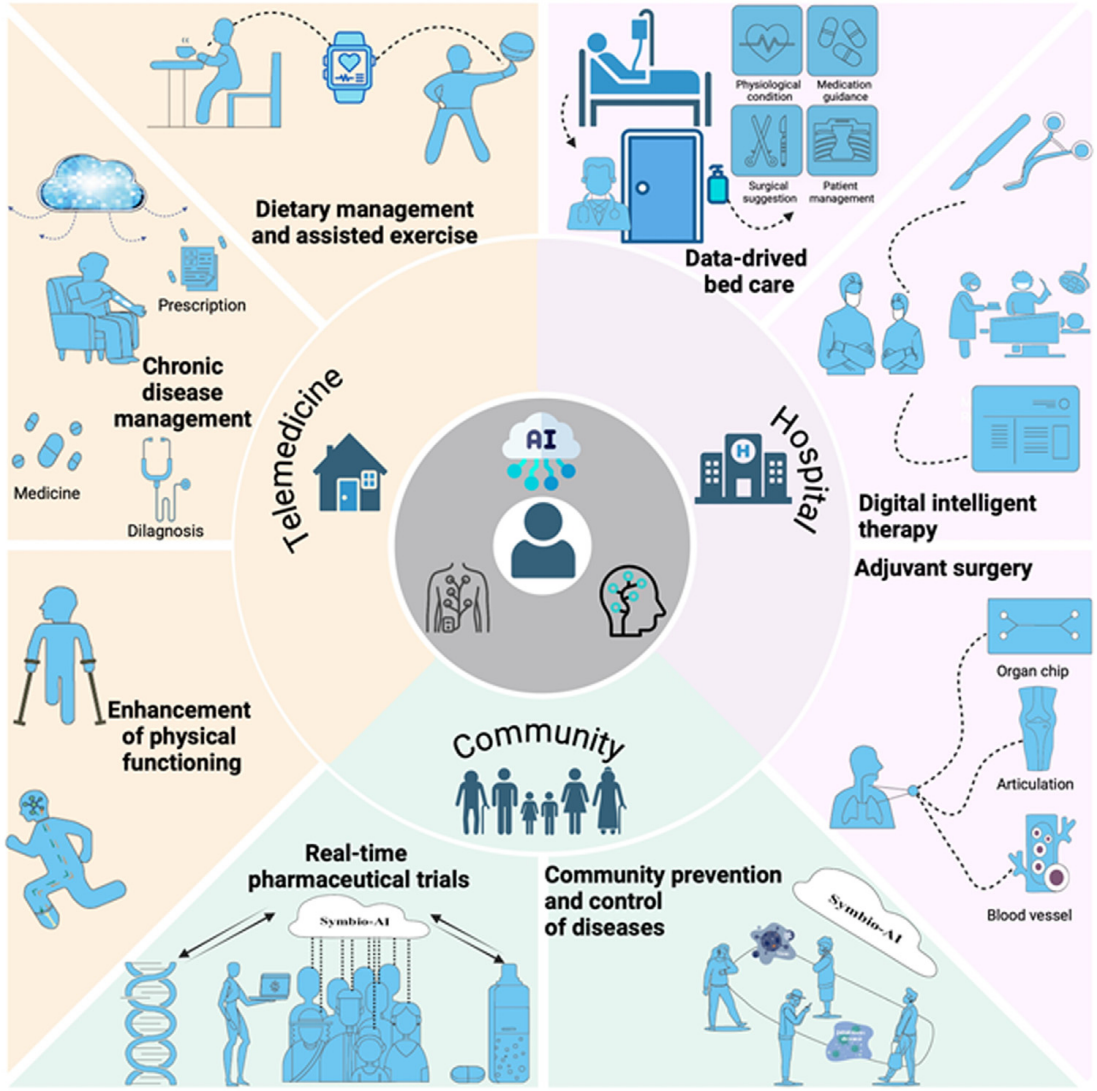
**Typical implementations of SymAI in personal healthcare and public health**.

The human-centered SymAI framework, equipped with AI cores and wearable or implantable devices, extends and enhances human body functions while fostering symbiotic interactions between humans and machines. This innovative framework aims to deeply integrate AI technologies into daily human activities, ensuring that individuals’ physiological and cognitive needs are effectively monitored and met through real-time adaptive responses. The network of SymAI devices, featuring high-level data aggregation and terminal analysis capabilities, functions as a cohesive system to support various healthcare applications. As shown in Fig. 2, SymAI demonstrates the role of smart healthcare in scenarios such as telemedicine, hospitals, and communities by integrating intelligent devices and systems.

In the past decades, telemedicine has been greatly empowered by smart sensor devices, in most cases embedded in mobile applications. AI-driven data insights can deliver advanced diagnostics and adaptive treatment modalities, enabling precision medicine and improving patient outcomes in chronic disease management at home [3]. Chronically ill patients may benefit from customized care settings, facilitated by real-time monitoring of physiological functions and automated therapeutic administration or physical therapies. The integration of intelligent furniture, ubiquitous computing devices, and edge-AI centers creates a semi-closed domestic environment focused on personalized health monitoring that is aimed at diagnosing and alleviating physical impairments. Human physiological signals can be accurately interpreted by various neural interfaces (brain-computer interfaces, high-density EMG sensor arrays, etc.), enabling the enhancement of physical functioning through the control of smart prostheses [4,5]. Individuals experiencing difficulties with emotional processing, as well as the elderly, can benefit from bespoke emotional support systems that provide companionship and emergency assistance, reducing the risk of dementia and accidental death. Furthermore, these intelligent systems can also provide users with personalized dietary recommendations and record users’ dietary habits. By combining AI algorithms, the system can analyze users’ real-time feedback, ensuring that the nutritional components they consume align with their health needs. Smart fitness devices can create personalized exercise prescriptions based on users’ health conditions, facilitating rehabilitation and enhancing physical fitness.

In hospitals, SymAI provides precise real-time monitoring and bed management for inpatients, offering greater convenience and efficiency in reducing the burden on nursing and medical staff. For patients requiring surgical treatment, surgeons utilize implantable biocompatible sensor arrays or dissolvable imaging capsules combined with artificial intelligence and surgical robots to assist in performing highly efficient and precise surgeries [4,5]. Furthermore, SymAI could be incorporated into cutting-edge biological innovations such as personalized artificial organs like 3D-printed bone implants or blood vessel grafts, when integrated with closed-loop functionality monitoring. By leveraging data-driven insights and advanced biofabrication technologies, this approach enables the regeneration of damaged tissues and the restoration of physiological functions, ultimately leading to a substantial improvement in overall quality of life. In the personal care of inpatients, AI-driven medical technology may potentially supplant traditional approaches in precision medicine, medication management systems, smart surgical support systems, and the customization of therapeutic environments [4].

Moreover, SymAI can significantly help optimize public health policies—including collaborative health networks, integrated health and social services, and the tracing of the origin and transmission of viruses and epidemic diseases. SymAI leverages advanced data analytics and artificial intelligence to gather and analyze vast amounts of health-related data from diverse sources. This includes information from hospitals, laboratories, community health organizations, and even social media platforms. By integrating these data streams, SymAI can provide real-time insights into the dynamics of disease spread, identifying hotspots and potential outbreak areas before they escalate into larger public health crises. This proactive approach enables health authorities to implement timely interventions, such as targeted vaccination campaigns or public health advisories, thereby mitigating the impact of infectious diseases on communities. Compared to the challenges of traditional pharmaceutical clinical trials, which are often lengthy, costly, and complex, SymAI enhances real-time pharmaceutical trials by continuously monitoring patient responses and side effects during community treatment processes. This immediate feedback loop allows researchers to make quick adjustments to trial protocols, ensuring that therapies are both safe and effective, ultimately accelerating the pace of regulatory approval and patient access.

In the near future, SymAI would also facilitate the construction of a communication bridge between humans, effectively reducing the barriers to interpersonal information exchange. This is achieved through the establishment of bidirectional interactive networks between individuals, utilizing identity recognition and proactive connection. The network can integrate disparate data sets and provide personalized recommendations through adaptive learning algorithms, thereby the inclusion of diverse intelligent nodes enhances surveillance capabilities.

## 3 Technological components and hardware enablers

**Fig. 3:**
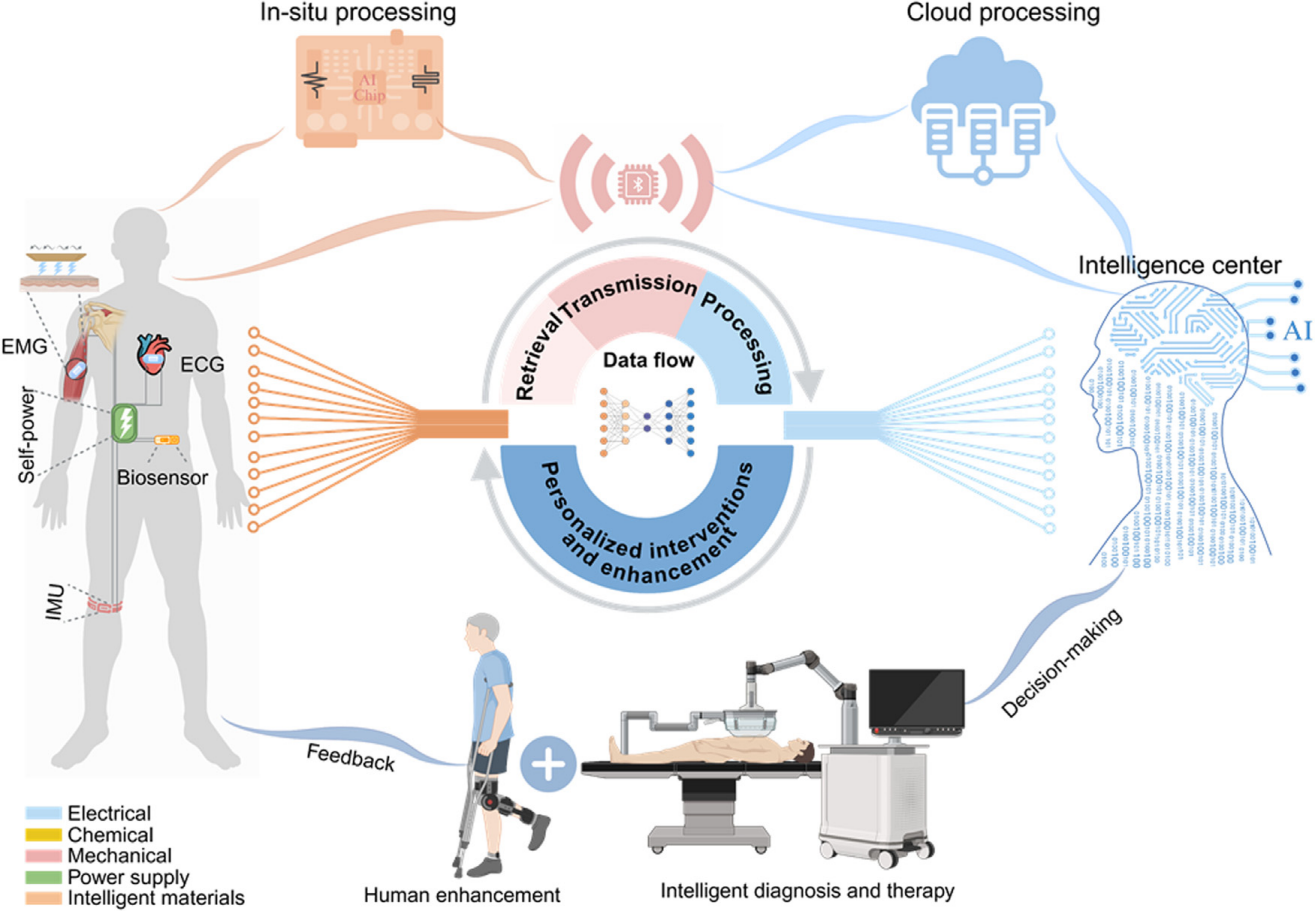
**Technological components and hardware enablers for SymAI**.

SymAI emphasizes the dynamic connection between humans and intelligent devices, with data playing a crucial role in this process. This section primarily outlines representative devices related to data flow across various application contexts. Fig. 3 illustrates the technological components and hardware enablers for establishing SymAI (Refer to Table 1 for representative technologies and products).

**Table 1:** **Representative technologies and products in the perspective**.

Technology/Product	Featured function	Year	Reference
MiniMed 670G	The first FDA-approved hybrid closed-loop system for people with Type 1 diabetes	2016	https://www.medtronic.com/ca-en/diabetes/home/products/insulin-pumps/minimed-670g.html
Zio XT Patch	Wearable ECG monitoring	2022	https://www.irhythmtech.com/providers/zio-service/zio-monitors
NeuroSky MindWave	Wearable EEG monitoring	2010	https://store.neurosky.com/pages/mindwave
Dexcom G7	Continuous glucose monitoring	2022	https://www.dexcom.com/en-CA/dexcom-g7-cgm-system
Gatorade Gx Patch	Sweat (sodium concentration) monitoring	2021	https://www.gatorade.com/gx-sweat-patch-how-to-use
Exo’s SweepAI	AI-powered ultrasonic medical imaging	2024	https://www.exo.inc/ai
Perception Neuron 3	High-precision motion monitoring and gait analysis	2016	https://neuronmocap.com/pages/perception-neuron-3
Microsoft HoloLens 2	A headset for mixed reality with Azure Kinect sensor, an ARM processor, eye-tracking sensors, etc.	2022	https://learn.microsoft.com/en-us/hololens/hololens2-hardware
Myo Armband (EMG device)	Intelligent gesture detection and human-computer interaction	2019	https://wearabletech.io/myo-bracelet/
Eccrine Systems’ sweat sensor	Advanced sweat sensor	2016	https://wearable-technologies.com/news/eccrine-systems-will-expand-r-d-capabilities-to-accelerate-development-of-wearable-sweat-sensors
Hexoskin Smart Shirt	Continuous cardiac, pulmonary, activity and sleep monitoring	2006	https://hexoskin.com
NearLink technology	New generation short-range wireless communication	2023	https://www.hisilicon.com/cn/techtalk/nearlink
5 G New Radio	Fifth-generation ultrafast wireless communication	2022	https://www.qualcomm.com/research/5g/5g-nr
IBM TrueNorth	Neurosynaptic processing	2015	https://research.ibm.com/publications/truenorth-design-and-tool-flow-of-a-65-mw-1-million-neuron-programmable-neurosynaptic-chip
Intel Loihi	Neuromorphic computing	2018	https://www.intel.com/content/www/us/en/research/neuromorphic-computing.html
BrainScaleS	Neuromorphic computing	2010	https://brainscales.kip.uni-heidelberg.de/index.html
SpiNNaker	Neuromorphic computing	2012	https://apt.cs.manchester.ac.uk/projects/SpiNNaker/
D-Wave Systems	Quantum computing	1999	https://www.dwavesys.com/company/about-d-wave/
IBM Q	Quantum computing	2016	https://www.ibm.com/quantum/technology
Google Pixel Buds Pro 2	Intelligent wireless audio device with Google Assistant	2024	https://blog.google/products/pixel/google-pixel-buds-pro-2/

### 3.1 Multi-modal data retrieval

Physiological indicators can be broadly divided into electro-physiological, chemical, and mechanical indicators, covering important biological signals from the musculoskeletal, respiratory, nervous, and endocrine systems. The primary task of SymAI devices is to extract and analyze these indicators. Monitoring nervous system signals, such as electroencephalogram (EEG), electrocardiogram (ECG), and electromyogram (EMG), has a long history [5,6]. With technological advancements, the devices have evolved from bulky and cumbersome equipment to today’s intelligent wearables. An artificial intelligence (AI)-enabled electrocardiogram (ECG) model is developed to identify echocardiographically determined diastolic dysfunction and increased filling pressure, which is a simple and promising tool to enhance the detection of diseases associated with diastolic dysfunction and increased diastolic filling pressure [7]. Combining AI with various physiological sensing technologies is currently the mainstream trend in wearable devices. There are also mature products in the market that combine AI and physiological sensing. For example, the Zio XT Patch is a wearable ECG patch that continuously monitors cardiac rhythms for up to 14 days, integrating AI algorithms that analyze ECG data to detect arrhythmias. NeuroSky MindWave Mobile is a portable EEG headset that can monitor users' brainwave signals in real-time and perform brainwave analysis, for brain training, relaxation, and cognitive enhancement. The Myo Armband is an EMG device that enables gesture control by monitoring users’ muscle signals.

Smart sensors for chemical physiological indicators, including multi-omics blood atlas, sweat and urine components, and breathomics, have also advanced. A person with type 1 diabetes (T1D) is dependent on insulin therapy, and needs to make frequent dosing decisions to achieve glycemic targets. Thus, closed-loop control systems have the potential to automate insulin therapy for diabetes management. A closed-loop control system, also known as an artificial pancreas, is a network of drug delivery feedback loops, the idea of which glucose control was first proposed in the 1960s [8]. The first fully integrated commercial artificial pancreas system was the MiniMed 670 G, which was approved for T1D management in 2016. Up to now, there are relatively mature intelligent devices for diabetes closed-loop management in the market. The Dexcom G7 is a continuous glucose monitoring system for closed-loop management of diabetes. Eccrine Systems’ sweat sensor is specifically designed to monitor chemical components in sweat, providing insights into exercise status, body fluid balance, and electrolyte levels for health management. The Gatorade Gx Sweat Patch is a wearable sweat sensor that measures sweat rate and sodium loss, offering real-time hydration assessment by analyzing sodium concentration in sweat through a skin-adhered patch connected to a mobile app.

Mechanical physiological indicators are exemplified by hemodynamics monitoring, such as dynamic blood pressure monitoring devices based on photoplethysmography or strain-sensing units [3,5]. The Hexoskin Smart Shirt is a comfortable and washable garment with integrated textile sensors, continuously tracking breathing rate and volume, heart rate, and activity levels, with data accessible in real-time via a mobile app for comprehensive health and performance insights. Exo’s SweepAI is an AI-powered ultrasound imaging solution developed to simplify and enhance lung and cardiac examinations. Key features include intelligent guidance for image acquisition and advanced algorithms for real-time interpretation, enabling clinicians to quickly obtain and analyze high-quality ultrasound images of the lungs and heart, thus improving diagnostic efficiency and accuracy at the point of care [9]. Monitoring movement status is crucial for patients with joint diseases. Intelligent gait analysis devices like the Perception Neuron 3 high-precision full-body motion capture suit have been developed for knee and ankle joints, providing effective medical data for patients with joint diseases.

Notably, SymAI devices emphasize multi-modal interconnection, integrating the extraction of these physiological signals within the same data acquisition system, enabling holistic monitoring of multiple indicators.

### 3.2 Data transmission and processing

The data retrieved through SymAI devices could be transmitted to intelligent terminals for further analysis and processing. The data transmission module is essential in this context, transitioning from cable transmission to high-throughput, high-speed wireless transmission methods. The most widely used modules in intelligent wearable devices currently are Bluetooth modules, which are advantageous in low power consumption, high throughput, and moderate transmission range [10]. StarFlash technology has a transmission speed of up to 10 Gbps and can achieve stable connections at distances of tens of meters. Compared to Bluetooth technology, it significantly improves data transmission efficiency and reliability. 5 G NR (New Radio) modules are applied in remote medical devices, industrial Internet of Things, and smart city sensors, providing ultra-high data rates (up to 20 Gbps) and ultra-low latency (1 ms), suitable for large-scale device connectivity. Due to the higher speed and lower latency of 5 G networks, doctors can remotely control surgical robots in real-time and perform precise operations with little delay [11].

Data processing is a core component of SymAI, comprising edge computing and cloud computing. IBM TrueNorth and Intel Loihi are neuromorphic chips inspired by human brains with strong parallel processing capabilities, low power consumption, and high computing efficiency. BrainScaleS and SpiNNaker are brain-inspired computing processors that support large-scale parallel processing, event-driven computing mode, high plasticity, and online learning. D-Wave Systems and IBM Q are quantum neural network processors that utilize quantum superposition for parallel computing, capable of handling complex optimization problems and suitable for specific types of machine learning tasks.

In the process of data transmission and processing, it is crucial to address the significant challenge of data standardization and interoperability among different datasets. In the previously mentioned SymAI system, the data collected by various devices may exhibit discrepancies, and these devices might originate from different manufacturers. Therefore, it is essential to standardize the collected data. One effective solution is to establish a uniform data format standard, ensuring that data from different devices can be stored and transmitted in a consistent format. For instance, common medical data formats include HL7 and DICOM. Alternatively, preprocessing the collected data, such as developing data conversion tools to transform data from different devices into a unified format, can ensure data interoperability. These two data standardization techniques, which are widely employed, effectively standardize data from diverse sources and enhance interoperability among data from different devices.

### 3.3 Personalized interventions and enhancement

The ultimate goal of SymAI is to aid humans by offering beneficial interventions or capability enhancement. In the management of chronic diseases, precise medication administration can be accomplished by sophisticated monitoring systems and advanced drug delivery systems like smart microneedle patches and nanocapsules [3,9]. During the surgical process, the physiological data provided by SymAI will assist doctors in making medical decisions, and intelligent surgical robots will complete the surgical tasks. In the field of organ transplantation, a series of artificial organs and tissues have been developed, such as artificial hearts, artificial kidneys, artificial joints, etc. Google Pixel Buds can enhance hearing and provide simultaneous translation for different languages [5]. These devices could also enhance sensory, physical, and communication abilities. For example, smart glasses like the Microsoft HoloLens 2 can enhance vision and intelligently analyze the visual environment, providing augmented reality experiences for both professional and personal applications. Smart shoes and clothing, such as the Digitsole Smart Shoes and Nadi X Yoga Pants, capture the wearer’s posture through body-sensing technology and provide corrective feedback to improve form and posture. Exoskeletons like the ReWalk Personal Exoskeleton and EksoNR assist with mobility and can correct abnormal posture through supportive exoskeleton frameworks utilizing advanced materials and sensors. Furthermore, various smart home products like the Amazon Echo, Google Nest Hub, and Philips Hue Smart Lighting systems also fall into this category, aiming to improve quality of life and work efficiency by enhancing the external capabilities of individuals [4,5,11].

In summary, hardware enablers for multimodal data retrieval, data transmission, and processing are invaluable for achieving the beneficial coexistence of humans and machines in our rapidly approaching, highly sophisticated future. These systems interact and cooperate with their human-interfaced counterparts through the interchange of vital data, providing beneficial solutions at both ends.

### 3.4 Outlook: The recruitment of flexible electronics and advanced materials

*Symbiopersonal Intelligence* is poised to transform the future of personal healthcare and public health through the convergence of flexible electronics and advanced materials. Flexible electronics, enabled by innovations in materials science, such as biocompatible conducting polymers, smart nanomaterials, and two-dimensional materials, offer lightweight, stretchable, and conformable sensors that seamlessly integrate with the human body, whether wearable or implantable, allowing for continuous and unobtrusive monitoring of physiological signals. Coupling flexible electronics with AI algorithms can analyze complex physiological data in real time, therefore facilitating predictive analytics, personalized diagnostics, and autonomous feedback systems. Moreover, the application of flexible powering systems in wearable technology is still in its infancy, necessitating further validation to ensure that energy density can meet safety requirements. Additionally, the processes of data transmission and processing within various sensing modules currently operate independently, indicating a significant gap between current sensing technologies and their integration with SymAI. Addressing these challenges highlights an important direction for future research efforts, as thoroughly discussed in other reviews [6,11,12]. By overcoming hurdles in material development, power supply, and system integration, and by fully integrating AI into sensor technologies, we can develop intelligent, self-adaptive healthcare systems through SymAI that not only monitor health conditions but also predict and prevent diseases. Ultimately, this convergence will lead to improved personal healthcare and enhanced public health outcomes.

A perceived major concern associated with AI in general and consequently will be associated with SymAI is that healthcare leaders believe it could be challenging to follow standardized care processes and ensure the security of health data versus privacy when AI systems are implemented in healthcare. Concerns arise regarding the uncertainties surrounding when an AI algorithm can be deemed “valid enough” and “truthful enough” for integration into standardized care processes. One major issue is the poor explainability of artificial intelligence, which is attributed to its complex structure, a large number of parameters, and the intricate interactions between features. In contrast, the SymAI algorithm addresses these challenges by employing interpretable methods, such as decision trees, and providing feature importance scores. This approach helps users understand which input features have the greatest impact on the model’s decisions, thereby enhancing their comprehension of the model's outputs. Additionally, SymAI utilizes visualization tools to illustrate the decision-making process, displaying data flows and decision paths through charts and graphics. This makes complex decision processes more intuitive. Furthermore, the algorithm incorporates a user feedback mechanism that allows users to question and provide input on the AI’s decisions, promoting continuous improvements in the model and enhancing transparency. Ensuring adequate security necessitates the use of advanced encryption and decentralized operations throughout data collection, transmission, and storage. Employing federal learning protects sample data security and user privacy by enabling model training without sharing raw data. Implementing robust and restricted access controls can further enhance security. These measures must comply with relevant data regulations—such as HIPAA, GDPR, and the Personal Information Security Code—to ensure that health data is handled in accordance with legal and ethical standards.

## Declaration of competing interest

The authors declare that they have no conflicts of interest in this work.
